# Construction of a predictive model for osteoporosis risk in men: using the IOF 1-min osteoporosis test

**DOI:** 10.1186/s13018-023-04266-7

**Published:** 2023-10-11

**Authors:** Kun Zhang, Min Wang, Weidong Han, Weihong Yi, Dazhi Yang

**Affiliations:** 1 Health Science Center, Shenzhen University, Shenzhen, 518061 Guangdong China; 2 Department of Spine Surgery, Huazhong University of Science and Technology Union Shenzhen Hospital (Nanshan Hospital), Shenzhen, 518052 Guangdong China; 3 Department of Pain, The 8th Affiliated Hospital of Sun Yat-Sen University, Shenzhen, 518033 Guangdong China

**Keywords:** Osteoporosis, IOF, Prediction model

## Abstract

**Objective:**

To construct a clinical prediction nomogram model using the 1-min IOF osteoporosis risk test as an evaluation tool for male osteoporosis.

**Methods:**

The 1-min test results and the incidence of osteoporosis were collected from 354 patients in the osteoporotic clinic of our hospital. LASSO regression model and multi-factor logistic regression were used to analyze the risk factors of osteoporosis in patients, and the risk prediction model of osteoporosis was established. Verify with an additional 140 objects.

**Results:**

We used logistic regression to construct a nomogram model. According to the model, the AUC value of the training set was 0.760 (0.704–0.817). The validation set has an AUC value of 0.806 (0.733–0.879). The test set AUC value is 0.714 (0.609–0.818). The calibration curve shows that its advantage is that the deviation correction curve of the nomogram model can maintain a good consistency with the ideal curve. In terms of clinical applicability, compared with the "total intervention" and "no intervention" schemes, the clinical net return rate of the nomogram model showed certain advantages.

**Conclusion:**

Using the 1-min osteoporosis risk test provided by IOF, we built a male osteoporosis risk prediction model with good prediction effect, which can provide greater reference and help for clinicians.

## Introductions

Osteoporosis is a chronic disease characterized by decreased bone density and deterioration of bone microstructure and is a global disease with high incidence [1]. Due to bone fragility and a higher risk of future fractures, osteoporosis is rapidly becoming a critical health issue today [2]. At the same time, osteoporosis is often ignored and irreversible, once it occurs, it will bring multiple burdens such as life ability, spiritual needs and economic costs to patients [3]. At present, the measurement of bone mineral density by dual-energy X-ray absorption method is currently recognized as the gold standard for the diagnosis of osteoporosis [4], but dual-energy X-ray method is expensive and should not be measured repeatedly in the short term [5]. As a result, several clinical risk assessment tools have been developed to assess the risk of osteoporosis [6]. However, these tools tend to favor women more, especially postmenopausal women, and ignore men or are less effective at predicting which men are [7, 8]. Although women have a higher risk of osteoporosis than men, the lifetime risk of a non-traumatic fracture after osteoporosis is estimated to be about 25% for a 60-year-old man [9, 10]. Men are also twice as likely as women to die in hospital after a hip fracture [11]. The 1-year mortality rate after fracture was 31% for men and 17% for women [12]. In addition, studies have shown that most older men with pre-existing fragility fractures do not know to take screening bone mineral density (BMD) tests or receive treatment [13]. Therefore, a convenient, simple and highly controllable screening method for men is needed to reflect the risk of osteoporosis in the body and provide a preventive basis for the prevention of osteoporosis in men [14]. The International Osteoporosis Foundation (IOF) offers a 1-min osteoporosis risk test, a simple and sensitive primary screening tool [15]. The survey is an internationally recognized tool for raising awareness and consists of 19 questions. Compared with the traditional evaluation of osteoporosis, the IOF questionnaire is a more convenient, simple, controllable and authoritative method, which can effectively replace the dual-energy X-ray method for bone mineral density detection in the population, thus reflecting the risk of osteoporosis in the body and providing a basis for follow-up detection. And further evaluation by a primary care physician [8, 16]. Therefore, this paper aims to construct a clinical prediction model for elderly people using 1-min osteoporosis risk test as an evaluation tool for osteoporosis and verify its accuracy and clinical practicability.

## Materials and methods

### Survey object

From January to June 2023, patients in the orthopedic osteoporosis clinic of Union Shenzhen Hospital of Huazhong University of Science and Technology (Nanshan Hospital) and Liwan Community Health Service Center of Shenzhen Nanshan Medical Group Headquarters were selected as the research objects to construct the prediction model. Inclusion criteria include (1) no secondary osteoporosis, (2) clear mind, no communication disorders, and (3) no long-term bed rest or no exposure to sunlight. Exclusion criteria include (1) patients with endocrine diseases such as type 2 diabetes mellitus; (2) patients with a history of chronic heart dysfunction, tumors, impaired liver and kidney function, and immune system diseases; (3) Patients with a history of surgery or disease that may cause gastrointestinal malabsorption; and (4) those who did not cooperate or did not complete the questionnaire.

### Research methods

A 1-min test of osteoporosis risk was performed on all subjects, while basic information such as age, gender, and BMI were recorded. Bone mineral density was measured by dual-energy X-ray method, and the bone mineral density of lumbar spine, spine and radius was recorded, and the lowest bone mineral density was selected for recording.

### Diagnostic criteria

The patients were diagnosed with osteoporosis according to the diagnostic criteria for Primary Osteoporosis (2022). Bone mineral density *T* value ≤ 2.5 indicates osteoporosis.

### Statistical methods

R language (R.4.1.1) was used for data analysis. We then randomly divided the data set constructed by the patients from Union Shenzhen Hospital (Nanshan Hospital) of Huazhong University of Science and Technology into a training set and a validation set at a ratio of 5:5 to construct and verify the prediction model. After that, patients from Liwan Community Health Service Center of Shenzhen Nanshan Medical Group Headquarters were used as test sets to improve the reliability and robustness of the study results. Measurement data are expressed as Median (IQR) and counting data are expressed as n (%). Mann–Whitney *U* test, Pearson Chi-square test or Fisher exact probability method were used for comparison between groups, respectively. LASSO regression analysis is implemented through the "glmnet package," the "rms" package for the drawing of the nomogram and calibration curves, the "pROC" package for the ROC curve, and the Area under the ROC Curve (AUC) for evaluating the judgment of the nomogram. The internal verification of the nomogram model was realized by Bootstrap self-sampling 1000 times. Calibration curves are used to assess the predictive consistency of a nomogram. *P* < 0.05 was considered to be statistically significant.

### Results

Of the 354 people who were eventually included in the training and test sets, 106 had osteoporosis. People with bone pine are older than those without osteoporosis. Having a parent diagnosed with osteoporosis or a hunchback in one of the parents; Smoking; Alcoholism; The proportion of patients with osteoporosis was greater in the problems such as rash fracture (*P* < 0.05). (See Table 1 for details).

**Table 1:** Whether the risk of developing osteoporosis is compared with the 1-min test

Variables	Levels	No osteoporosis (*n* = 248)	Osteoporosis 1 (*n* = 106)	*P*
Age (years)	Median (IQR)	61.0 (57.0 to 64.0)	62.0 (60.0 to 65.0)	0.001
BMI (kg/m^2^)	Median (IQR)	23.8 (22.0 to 25.8)	23.4 (21.3 to 25.5)	0.231
Have either of your parents been diagnosed with osteoporosis or broken a bone after a minor fall (a fall from standing height or less)?	No	206 (83.1%)	63 (59.4%)	< 0.001
	Yes	42 (16.9%)	43 (40.6%)	
Did either of your parents have a stooped back (dowager’s hump)?	No	196 (79%)	57 (53.8%)	< .0001
	Yes	52 (21%)	49 (46.2%)	
Are you 40 years old or older?	No	29 (11.8%)	5 (4.7%)	0.062
	Yes	217 (88.2%)	101 (95.3%)	
Have you ever broken a bone after a minor fall, as an adult?	No	202 (81.5%)	64 (60.4%)	< 0.001
	Yes	46 (18.5%)	42 (39.6%)	
Do you fall frequently (more than once in the last year) or do you have a fear of falling because you are frail?	No	218 (87.9%)	69 (65.1%)	< 0.001
	Yes	30 (12.1%)	37 (34.9%)	
After the age of 40, have you lost more than 3 cm in height (just over 1 inch)?	No	192 (77.4%)	48 (45.3%)	< 0.001
	Yes	56 (22.6%)	58 (54.7%)	
Are you underweight (is your Body Mass Index less than 19 kg/m2)?	No	230 (92.7%)	69 (65.1%)	< 0.001
	Yes	18 (7.3%)	37 (34.9%)	
Have you ever taken corticosteroid tablets (cortisone, prednisone, etc.) for more than three consecutive months?	No	230 (92.7%)	77 (72.6%)	< 0.001
	Yes	18 (7.3%)	29 (27.4%)	
Have you ever been diagnosed with rheumatoid arthritis?	No	226 (91.1%)	71 (67%)	< 0.001
	Yes	22 (8.9%)	35 (33%)	
Have you been diagnosed with an over-active thyroid, overactive parathyroid glands, type 1 diabetes or a nutritional/gastrointestinal disorder such as Crohn’s or celiac disease?	No	226 (91.1%)	72 (67.9%)	< 0.001
	Yes	22 (8.9%)	34 (32.1%)	
Have you ever suffered from impotence, lack of libido or other symptoms related to low testosterone levels?	No	196 (79%)	86 (81.1%)	0.760
	Yes	52 (21%)	20 (18.9%)	
Do you regularly drink alcohol in excess of safe drinking limits (more than two units a day)?	No	219 (88.3%)	79 (74.5%)	0.002
	Yes	29 (11.7%)	27 (25.5%)	
Do you currently, or have you ever, smoked cigarettes?	No	130 (52.4%)	33 (31.1%)	< 0.001
	Yes	118 (47.6%)	73 (68.9%)	
Is your daily level of physical activity less than 30 min per day (housework, gardening, walking, running, etc.)?	No	164 (66.1%)	51 (48.1%)	0.002
	Yes	84 (33.9%)	55 (51.9%)	
Do you avoid, or are you allergic to milk or dairy products, without taking any calcium supplements?	No	186 (75%)	59 (55.7%)	< 0.001
	Yes	62 (25%)	47 (44.3%)	
Do you spend less than ten minutes per day outdoors (with part of your body exposed to sunlight), without taking vitamin D supplements?	No	207 (83.5%)	62 (58.5%)	< .0001
	Yes	41 (16.5%)	44 (41.5%)	

We randomly divided the study population into a training set (*n* = 177) and a validation set (*n* = 177) according to the osteoporosis rate at a ratio of 1:1. There was no significant difference in the 1-min test results between the two datasets (P > 0.05). (See Table 2 for details).

**Table 2:** A comparison of 1-min tests of osteoporosis risk in training sets and validation sets

Variables	Levels	Training set (*n* = 177)	Validation set (*n* = 177)	*P*
Age (years)	Median (IQR)	61.00 (57.00 to 64.00)	61.00 (58.00 to 64.00)	0.603
BMI (kg/m^2^)	Median (IQR)	23.53 (21.80 to 25.73)	23.88 (22.04 to 25.59)	0.537
Have either of your parents been diagnosed with osteoporosis or broken a bone after a minor fall (a fall from standing height or less)?	No	137 (77.4%)	132 (74.6%)	0.619
	Yes	40 (22.6%)	45 (25.4%)	
Did either of your parents have a stooped back (dowager’s hump)?	No	135 (76.3%)	118 (66.7%)	0.060
	Yes	42 (23.7%)	59 (33.3%)	
Are you 40 years old or older?	No	15 (8.6%)	19 (10.7%)	0.613
	Yes	160 (91.4%)	158 (89.3%)	
Have you ever broken a bone after a minor fall, as an adult?	No	135 (76.3%)	131 (74%)	0.712
	Yes	42 (23.7%)	46 (26%)	
Do you fall frequently (more than once in the last year) or do you have a fear of falling because you are frail?	No	147 (83.1%)	140 (79.1%)	0.416
	Yes	30 (16.9%)	37 (20.9%)	
After the age of 40, have you lost more than 3 cm in height (just over 1 inch)?	No	128 (72.3%)	112 (63.3%)	0.088
	Yes	49 (27.7%)	65 (36.7%)	
Are you underweight (is your Body Mass Index less than 19 kg/m^2^)?	No	152 (85.9%)	147 (83.1%)	0.557
	Yes	25 (14.1%)	30 (16.9%)	
Have you ever taken corticosteroid tablets (cortisone, prednisone, etc.) for more than three consecutive months?	No	156 (88.1%)	151 (85.3%)	0.531
	Yes	21 (11.9%)	26 (14.7%)	
Have you ever been diagnosed with rheumatoid arthritis?	No	152 (85.9%)	145 (81.9%)	0.386
	Yes	25 (14.1%)	32 (18.1%)	
Have you been diagnosed with an over-active thyroid, overactive parathyroid glands, type 1 diabetes or a nutritional/gastrointestinal disorder such as Crohn’s or celiac disease?	No	152 (85.9%)	146 (82.5%)	0.466
	Yes	25 (14.1%)	31 (17.5%)	
Have you ever suffered from impotence, lack of libido or other symptoms related to low testosterone levels?	No	140 (79.1%)	142 (80.2%)	0.895
	Yes	37 (20.9%)	35 (19.8%)	
Do you regularly drink alcohol in excess of safe drinking limits (more than two units a day)?	No	155 (87.6%)	143 (80.8%)	0.109
	Yes	22 (12.4%)	34 (19.2%)	
Do you currently, or have you ever, smoked cigarettes?	No	78 (44.1%)	85 (48%)	0.522
	Yes	99 (55.9%)	92 (52%)	
Is your daily level of physical activity less than 30 min per day (housework, gardening, walking, running, etc.)?	No	110 (62.1%)	105 (59.3%)	0.663
	Yes	67 (37.9%)	72 (40.7%)	
Do you avoid, or are you allergic to milk or dairy products, without taking any calcium supplements?	No	116 (65.5%)	129 (72.9%)	0.167
	Yes	61 (34.5%)	48 (27.1%)	
Do you spend less than ten minutes per day outdoors (with part of your body exposed to sunlight), without taking vitamin D supplements?	No	137 (77.4%)	132 (74.6%)	0.619
	Yes	40 (22.6%)	45 (25.4%)	

**Fig. 1:**
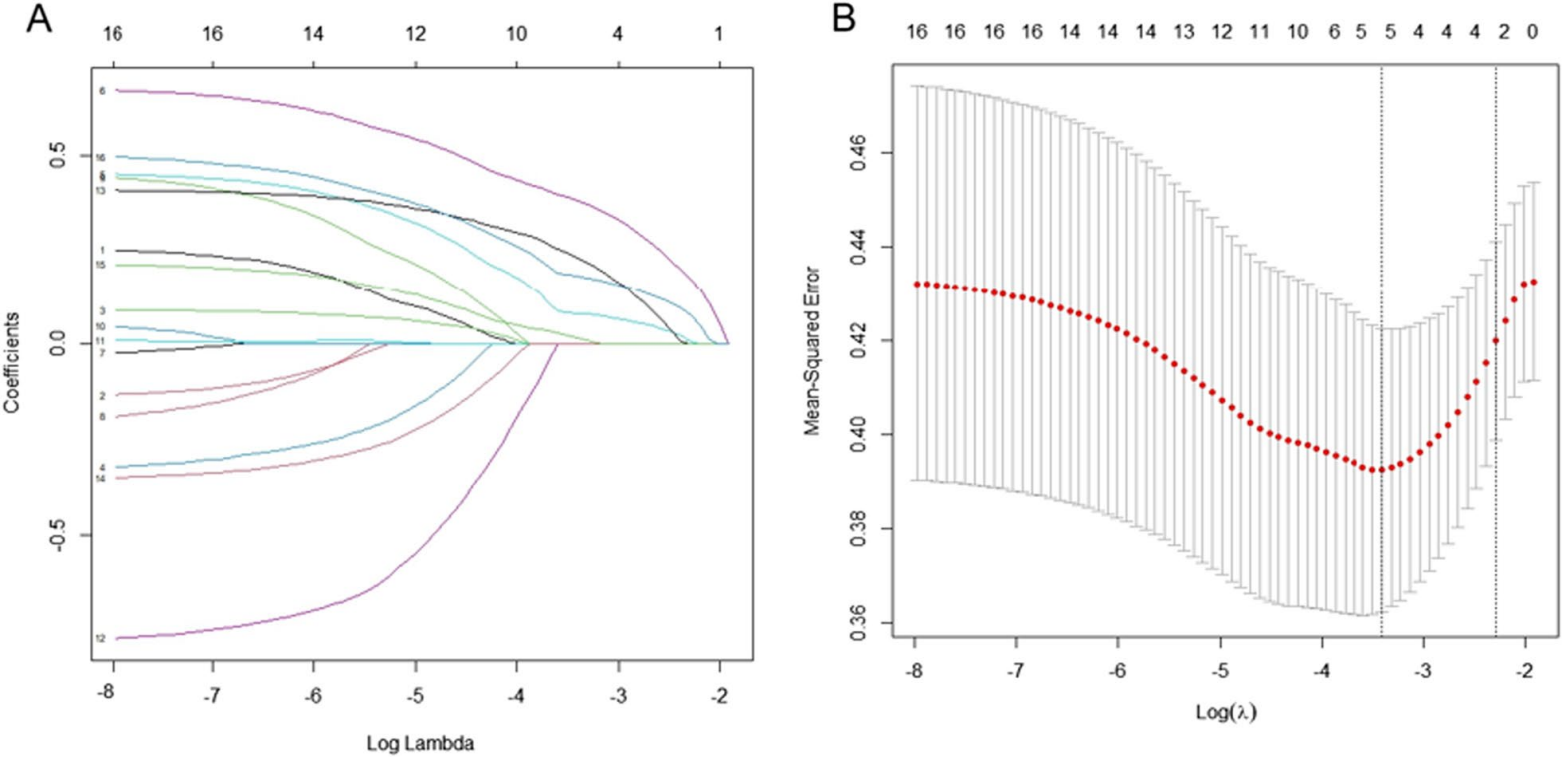
Texture feature selection using the Minimum Absolute shrink and selection operator (LASSO) binary logistic regression model. **A** The optimal penalty coefficient lambda (*λ*) was identified in the LASSO model, and 10 × cross-validation 140/90 was performed in the group. **B** The LASSO coefficient profiles of 21 features in the group were observed in the LASSO coefficient profiles as *λ* of the LASSO algorithm changed

The variable has a nonzero coefficient. The questions included five questions, including Are you underweight; Are you underweight (is your Body Mass Index less than 19 kg/m^2^); After the age of 40, have you lost more than 3 cm in height; Do you currently, or have you ever, smoked cigarettes; Do you spend less than ten minutes per day outdoors (with part of your body exposed to sunlight), without taking vitamin D supplements (see Fig. 1 for details).

Five characteristic variables selected from the LASSO regression model were included in the multifactor logistic regression, and the results showed that age (OR: 1.05; 95% CI 1.02–1.09); Are you underweight (OR: 3.32; 95% CI 1.51–7.39); After the age of 40, have you lost more than 3 cm in height(OR: 2.58; 95% CI 1.41–4.74); Do you currently, or have you ever, smoked cigarettes(OR: 1.93; 95% CI 1.13–3.33 was an independent risk factor for osteoporosis. (See Table 3 for details).

**Table 3:** Logistic regression analysis of risk factors for osteoporosis

Variables	OR1	95% CI	P
Age (years)	1.05	1.02, 1.09	0.004
Are you underweight (is your Body Mass Index less than 19 kg/m^2^)?			
No	–	–	
Yes	3.32	1.51, 7.39	0.003
After the age of 40, have you lost more than 3 cm in height (just over 1 inch)?			
No	–	–	
Yes	2.58	1.41, 4.74	0.002
Do you currently, or have you ever, smoked cigarettes?			
No	–	–	
Yes	1.93	1.13, 3.33	0.017
Do you avoid, or are you allergic to milk or dairy products, without taking any calcium supplements?			
No	–	–	
Yes	0.68	0.34, 1.31	0.300
Do you spend less than ten minutes per day outdoors (with part of your body exposed to sunlight), without taking vitamin D supplements?			
No	–	–	
Yes	1.52	0.76, 2.97	0.200

*1OR* odds ratio, CI confidence interval

**Fig. 2:**
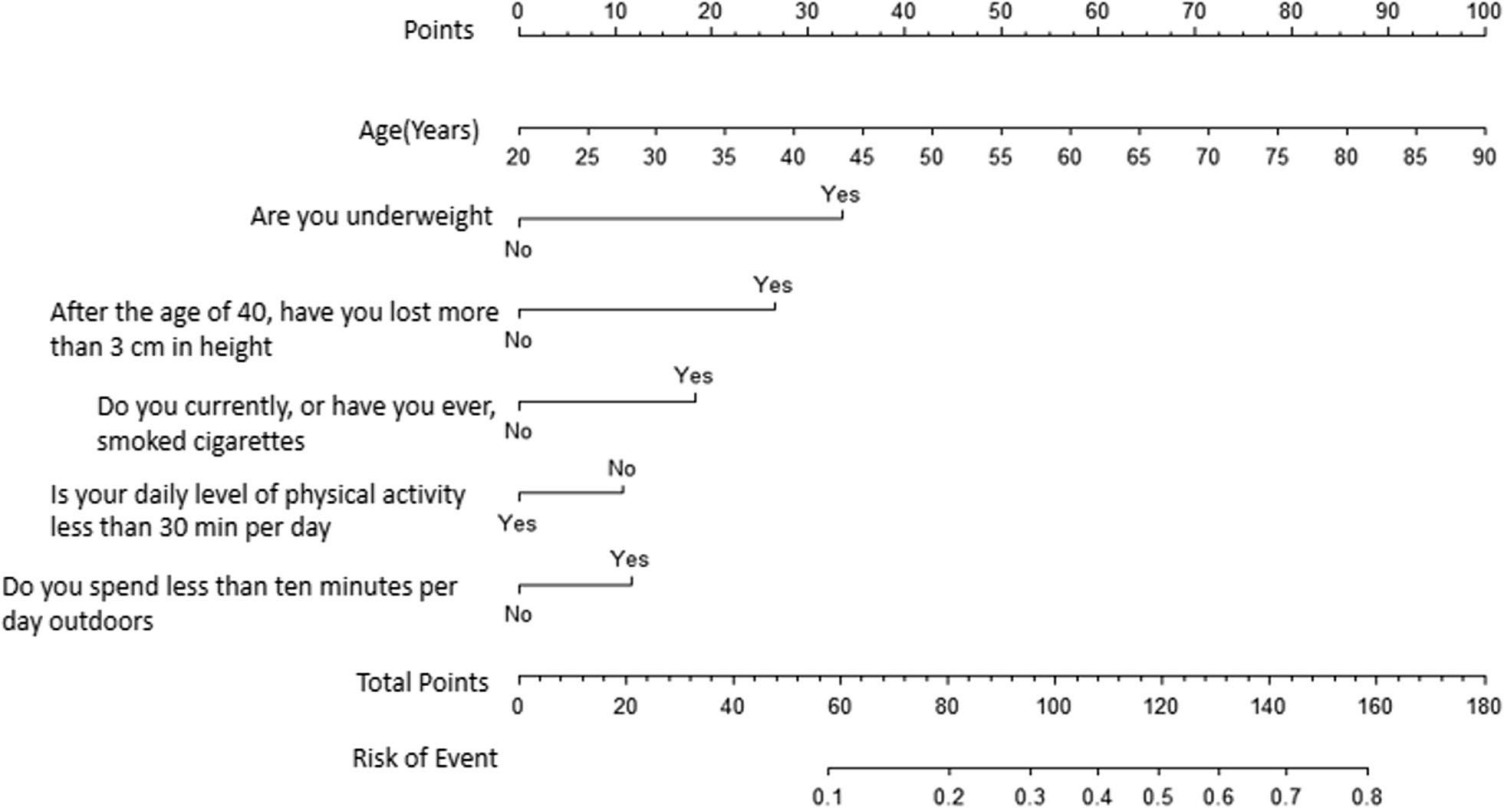
A graph predicting osteoporosis risk

A nomogram prediction model of osteoporosis was established based on logistic regression model. The model has a perfect score of 180, and when the score is more than 160, the risk of osteoporosis is 80%. (See Fig. 2 for details).

**Fig. 3:**
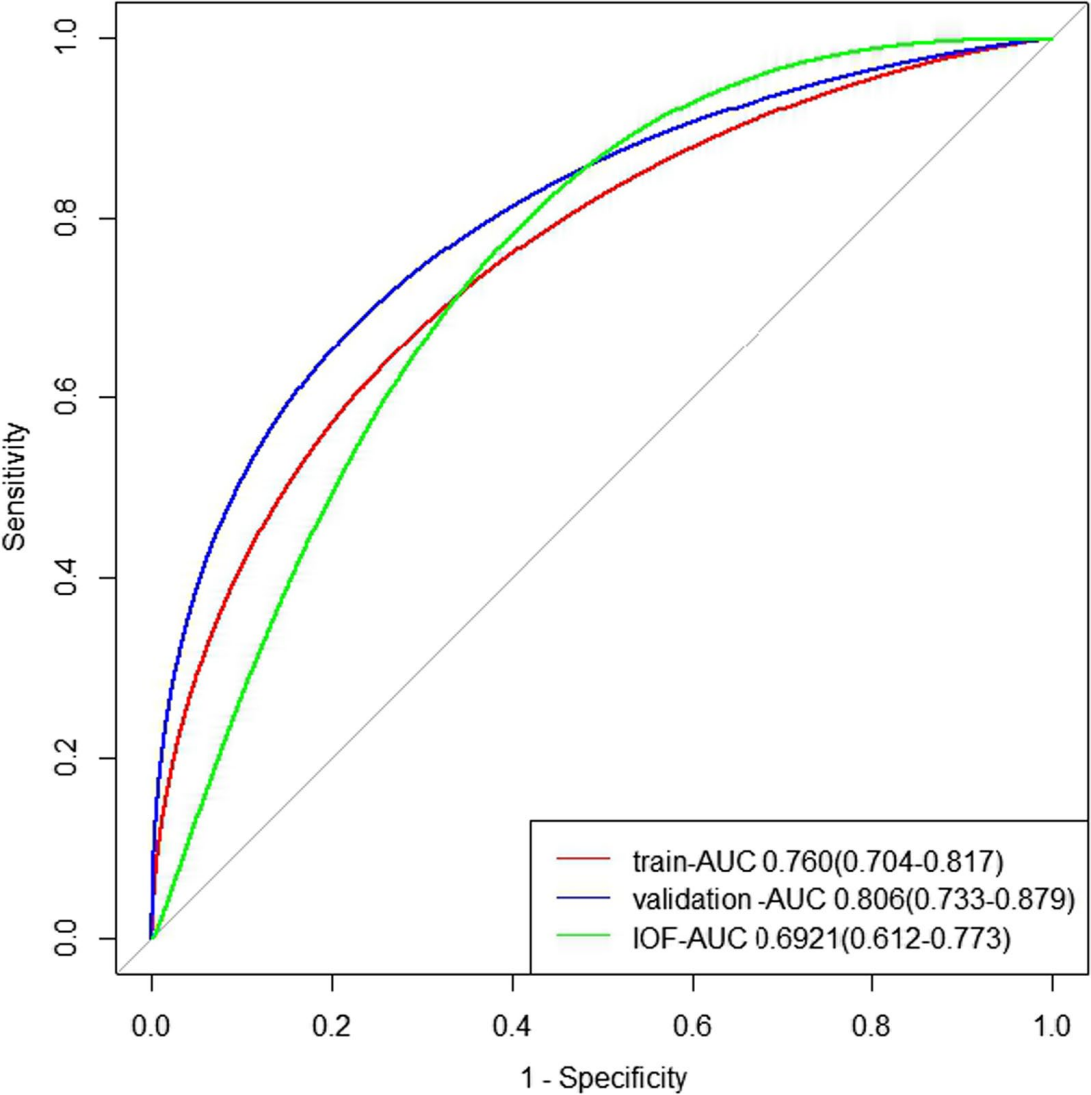
Receiver operating characteristic curve of the nomogram. AUC, area under curve

Meanwhile, according to the model, the AUC value of the training set is 0.760 (0.704–0.817). The validation set has an AUC value of 0.806 (0.733–0.879). In addition, we calculated the AUC of the test results according to the judgment criteria of the IOF 1-min test questionnaire, and the result showed that the AUC value of 0.692 (0.612–0.773) was lower than our prediction model. (See Fig. 3 for details).

**Fig. 4:**
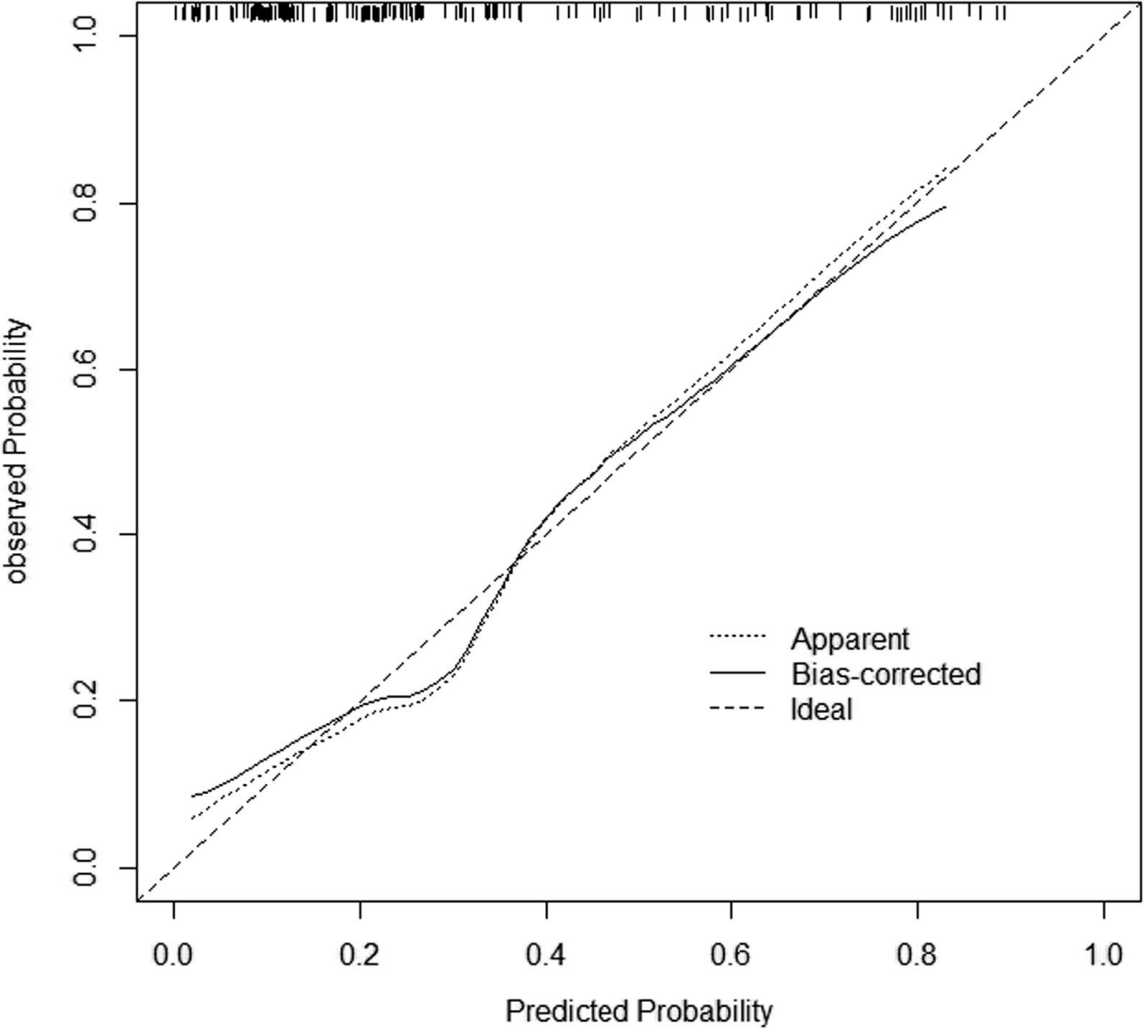
Calibration curve of the prediction nomogram

The calibration curves of the nomogram prediction model were verified internally by bootstrap resampling 1000 times. The results showed that the nomogram model had good calibration degree and prediction consistency. (See Fig. 4 for details).

**Fig. 5:**
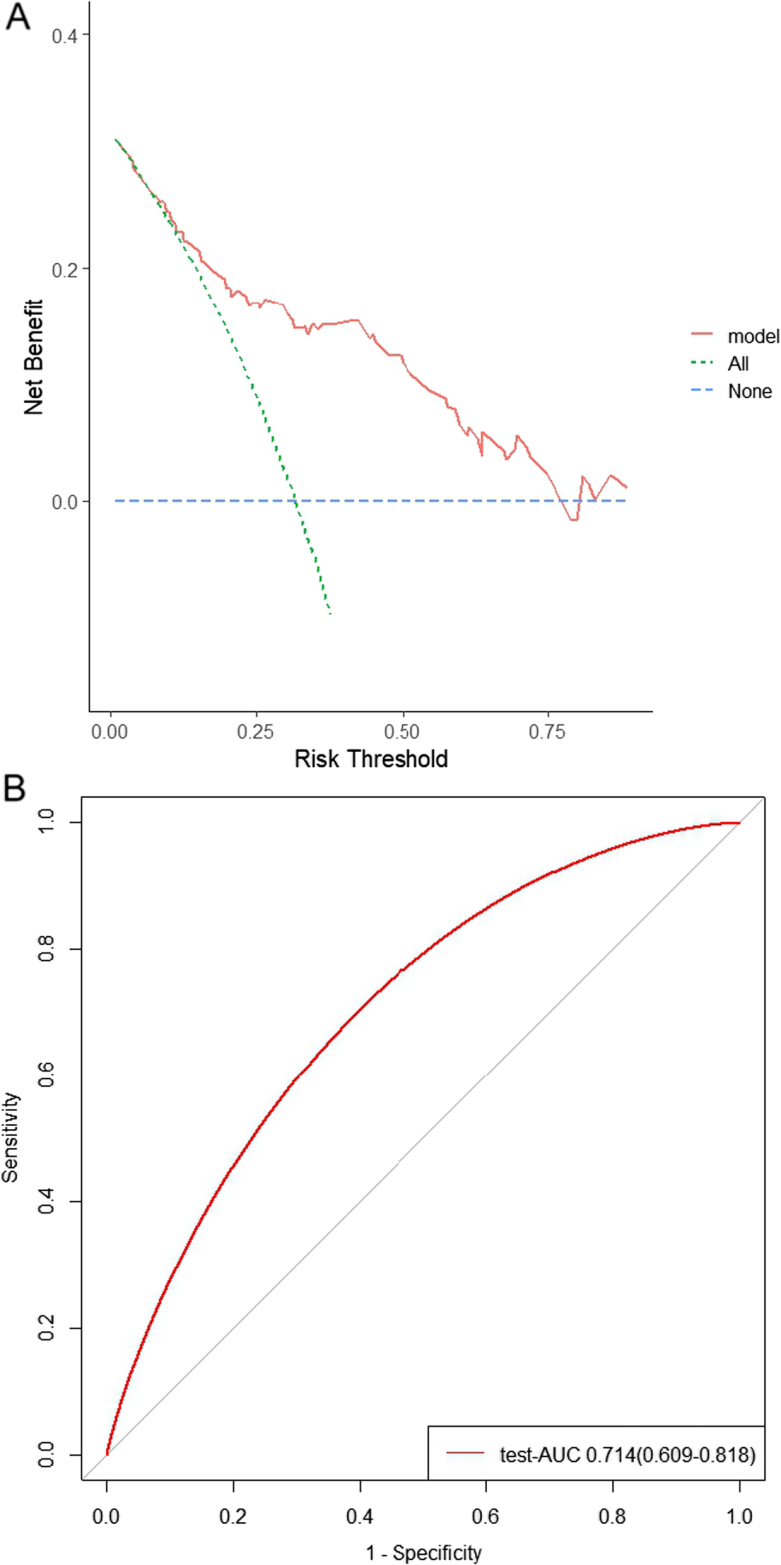
**a** DCA curve of the prediction model. **b** ROC curve of the test set

According to the DCA curve, when the prediction probability threshold of the nomogram model is 0–0.75, the clinical net return rate of the nomogram model is greater than that of the "full intervention" and "no intervention" schemes, suggesting that the nomogram model has good clinical applicability. (See Fig. 5 for details).

We then put our model to the final test using patients from another community health service center as a test set. A total of 140 patients were collected; detailed information is shown in Table 4.

**Table 4:** Comparison of the one-minute test on the risk of osteoporosis among 140 subjects in the test set

Variables	Levels	No osteoporosis (*n* = 98)	Osteoporosis 1 (*n* = 42)	*P*
Age (years)	Median (IQR)	23.8 (22.0 to 26.2)	23.4 (21.6 to 25.2)	0.571
BMI (kg/m^2^)	Median (IQR)	60.0 (56.0 to 63.0)	62.0 (60.0 to 64.0)	0.015
Have either of your parents been diagnosed with osteoporosis or broken a bone after a minor fall (a fall from standing height or less)?	No	82 (83.7%)	24 (57.1%)	0.002
	Yes	16 (16.3%)	18 (42.9%)	
Did either of your parents have a stooped back (dowager’s hump)?	No	80 (81.6%)	21 (50%)	< 0.001
	Yes	18 (18.4%)	21 (50%)	
Are you 40 years old or older?	No	12 (12.2%)	2 (4.8%)	0.296
	Yes	86 (87.8%)	40 (95.2%)	
Have you ever broken a bone after a minor fall, as an adult?	No	81 (82.7%)	24 (57.1%)	0.003
	Yes	17 (17.3%)	18 (42.9%)	
Do you fall frequently (more than once in the last year) or do you have a fear of falling because you are frail?	No	88 (89.8%)	28 (66.7%)	0.002
	Yes	10 (10.2%)	14 (33.3%)	
After the age of 40, have you lost more than 3 cm in height (just over 1 inch)?	No	79 (80.6%)	21 (50%)	< 0.001
	Yes	19 (19.4%)	21 (50%)	
Are you underweight (is your Body Mass Index less than 19 kg/m2)?	No	95 (96.9%)	27 (64.3%)	< 0.001
	Yes	3 (3.1%)	15 (35.7%)	
Have you ever taken corticosteroid tablets (cortisone, prednisone, etc.) for more than three consecutive months?	No	91 (92.9%)	29 (69%)	< 0.001
	Yes	7 (7.1%)	13 (31%)	
Have you ever been diagnosed with rheumatoid arthritis?	No	88 (89.8%)	27 (64.3%)	< 0.001
	Yes	10 (10.2%)	15 (35.7%)	
Have you been diagnosed with an over-active thyroid, overactive parathyroid glands, type 1 diabetes or a nutritional/gastrointestinal disorder such as Crohn’s or celiac disease?	No	93 (94.9%)	26 (61.9%)	< .001
	Yes	5 (5.1%)	16 (38.1%)	
Have you ever suffered from impotence, lack of libido or other symptoms related to low testosterone levels?	No	73 (74.5%)	34 (81%)	0.543
	Yes	25 (25.5%)	8 (19%)	
Do you regularly drink alcohol in excess of safe drinking limits (more than two units a day)?	No	88 (89.8%)	32 (76.2%)	0.065
	Yes	10 (10.2%)	10 (23.8%)	
Do you currently, or have you ever, smoked cigarettes?	No	53 (54.1%)	13 (31%)	0.020
	Yes	45 (45.9%)	29 (69%)	
Is your daily level of physical activity less than 30 min per day (housework, gardening, walking, running, etc.)?	No	60 (61.2%)	22 (52.4%)	0.432
	Yes	38 (38.8%)	20 (47.6%)	
Do you avoid, or are you allergic to milk or dairy products, without taking any calcium supplements?	No	77 (78.6%)	23 (54.8%)	0.008
	Yes	21 (21.4%)	19 (45.2%)	
Do you spend less than ten minutes per day outdoors (with part of your body exposed to sunlight), without taking vitamin D supplements?	No	79 (80.6%)	23 (54.8%)	0.003
	Yes	19 (19.4%)	19 (45.2%)	

Inclusion of test set patients in our predictive model resulted in an AUC value of 0.714 (0.609–0.818), indicating that our model predicted well.

## Discussion

We developed a clinical prediction model for male osteoporosis using the IOF 1-min Osteoporosis Test Questionnaire, which was validated and tested to show that the model has good efficacy and clinical applicability. This fills the gap that has long been lacking a reliable and easy-to-implement screening tool to identify people at high risk of osteoporosis in older men [17].

Osteoporosis screening and risk assessment enable clinicians to determine which populations require follow-up interventions to reduce their risk of complications and death [18]. Consistent with other studies, our study found that age is the most important factor in osteoporosis, with older people at greater risk of osteoporosis [19]. Secondly, family history, fracture history, and height loss increase the risk of osteoporosis, which is similar to many clinical observations, and some studies have shown that a variety of factors, including family history, history of fractures, smoking, excessive alcohol consumption, rheumatoid arthritis, etc., are risk factors for osteoporosis [20]. Another risk factor associated with osteoporosis is BMI, which has been shown to be higher in heavier weight and slower in bone mass loss at the same height level [21].

Based on these risk factors in the osteoporosis risk 1-min test, we constructed a clinical risk prediction model after screening the characteristic variables using LASSO regression. The advantage of the nomogram used in this study is that its analysis results are more intuitive and effective. In developing the most appropriate model, we also included age due to the irreplaceability between age and the osteoporosis association. After that, a simple, inexpensive and effective preliminary screening tool was built to serve the risk factor prediction model in the later stage. In addition, rather than a 1-min risk test designed to help people become aware of their risk factors, our model is designed to assess the risk of osteoporosis [15]. The ROC of this model is 0.760 (0.704–0.817). This has a similar predictive effect compared to other predictive models, but it requires fewer problems and is easier to operate [7, 22]. Another advantage of our study is that we not only divided the original data set of our hospital into a training set and a test set to build and test the model, but also collected patients from other hospitals to verify the model. This makes our model more reliable.

Machine learning-based computing methods are becoming increasingly prominent in healthcare applications. While traditional statistical methods rely on inferencing relationships between variables, machine learning is able to predict a patient's status based on other information about the patient [23]. A review of 89 studies suggests that ML has the potential to be used to identify factors associated with the risk of osteoporosis, thereby predicting osteoporosis [24]. There are a variety of ML methods used, such as SVM, ANN, and random forest. The best performing and most popular models are SVM and logistic regression [25]. In addition, some deep learning models are widely used, and the best reported performance is nearly perfect. But more complex models require rich data sets to make modeling predictions useful; this is especially true of deep learning (neural network) models [26, 27]. A wide variety of features have been explored, and most studies related to the use of ML to diagnose osteoporosis have used imaging tests to constitute the algorithm's most important predictors, with X-rays, ultrasound, MRI imaging, and machine learning all applying the results to infer bone health. Compared with these more complex machine learning methods, although the prediction efficiency is slightly lower, our problem input is simpler, including only 6 variables such as age. At the same time, the way we use the bar chart also makes the prediction more intuitive [28, 29].

In terms of model stability, 1000 Bootstrap resampling shows that the model is stable and has good correction accuracy and prediction consistency. In terms of clinical applicability, compared with the "full intervention" and "no intervention" schemes, the prognosis of patients is better and the clinical benefits are higher, so it shows that the nomogram model used in this study has better clinical applicability. At the same time, in order to obtain the risk factors of osteoporosis patients, this study incorporated the clinical data of patients during hospitalization into the research model for analysis, which was easy to obtain. In summary, we use the 1-min test of osteoporosis risk provided by IOF to construct a risk prediction model with good prediction effect.

Here's the strength of the study: First, it is a study specifically looking at the prediction of osteoporosis in men. Second, we conducted a cross-sectional study rather than a retrospective study. In addition, we not only verified the developed model, but also selected another hospital to verify the model. We also implemented strict inclusion and exclusion criteria to eliminate selection bias as much as possible. The limitations were that the age range we included did not include the entire age group and was not validated in more other geographies, and secondly, we did not take into account more basic information such as patient income and education level.

## Conclusions

We developed a clinical predictive model for osteoporosis in men that was validated and tested to show good predictive outcomes.

Compared with other studies, our predictive model can effectively predict osteoporosis using basic questionnaire information without using imaging data. It compensates for the disadvantages of time-consuming and expensive traditional tests, as well as other complex machine learning algorithms that require more information, and can easily and quickly predict osteoporosis. The study may have implications for developing a possible diagnosis of osteoporosis and could be valuable for doctors screening patients for osteoporosis in primary hospitals or community health centers.

## Data Availability

The datasets used and/or analyzed during the current study are available from the corresponding author on reasonable request.
